# Initial symptoms of patients with coronavirus disease 2019 in Japan: A descriptive study

**DOI:** 10.1002/jgf2.378

**Published:** 2020-09-26

**Authors:** Junpei Komagamine, Taku Yabuki

**Affiliations:** ^1^ Department of Internal Medicine National Hospital Organization Tochigi Medical Center Utsunomiya Japan

**Keywords:** Coronavirus disease 2019, epidemiology, initial symptoms

## Abstract

Fever, cough, malaise, and sore throat are the most common initial symptoms of coronavirus disease 2019 (COVID‐19). However, no studies have ever been conducted to investigate the initial symptoms of COVID‐19 in Japan. By using publicly available data, we investigated 707 consecutive COVID‐19 patients who were diagnosed in 10 prefectures of Japan prior to May 16, 2020. The primary outcomes were the initial symptoms on the day of symptom onset. The proportions of patients with each symptom among the symptomatic patients were calculated. Of all the patients, 79 (11.2%) were asymptomatic. Among the 628 symptomatic patients, the most common initial symptom was fever (65.9%), followed by cough (23.5%), malaise (23.5%), and sore throat (12.9%). At least one of these four symptoms was reported in 88.2% of all symptomatic patients. Nineteen patients (3.0%) reported gastrointestinal symptoms without respiratory symptoms, while six patients (1.0%) reported only the loss of smell or taste as the initial symptom. As in other countries, the most common initial symptoms of COVID‐19 in Japan are fever, cough, malaise, and sore throat. Gastrointestinal symptoms without respiratory symptoms and the loss of smell and taste are uncommon initial symptoms in Japan.

## Graphical abstract

No studies have been conducted to investigate the clinical features of COVID‐19 in Japan. Therefore, the aim of our study was to determine the initial symptoms of COVID‐19 patients in Japan. The most common initial symptoms of COVID‐19 patients were fever, cough, malaise, or throat pain in Japan, like other countries

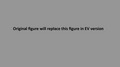

## 1 INTRODUCTION

In China, the first case of severe acute respiratory syndrome coronavirus 2 (SARS‐CoV‐2) was confirmed in December 2019.^1^ Since then, the number of coronavirus disease 2019 (COVID‐19) cases has increased to more than four million, and 0.3 million patients have died worldwide.^2^ In Japan, the number of patients with COVID‐19 has also reached more than 16 thousand as of June 1, 2020. To prevent the spread of COVID‐19, it is important to know the initial symptoms of COVID‐19.

Initial studies reported that most patients presented with fever and cough.^3^, ^4^, ^5^ Subsequent studies revealed that the frequencies of fever and cough as initial symptoms were not as high as initially thought,^6^, and atypical presentations including gastrointestinal symptoms, dysgeusia, and anosmia ere not uncommon.^7^, ^8^ However, few studies have focused on initial symptoms^5^ rather than symptoms reported at admission or during hospitalization.^3^, ^4^, ^6^, ^7^, ^8^ Furthermore, no studies have ever investigated the clinical features of COVID‐19 in Japan. Therefore, we evaluated the initial symptoms of COVID‐19 patients in Japan.

## 2 METHODS

### 2.1 Study design and setting

Japan consists of 47 prefectures. A descriptive study was conducted by analyzing publicly available data from ten prefectures located in an eastern part of Japan (supplementary file: Figure S1 and Table S1). These prefectures report all COVID‐19 cases confirmed by SARS‐CoV‐2 polymerase chain reaction (PCR) testing. These prefectures were chosen because detailed information on initial symptoms was documented in reports from each prefecture. All patients with confirmed COVID‐19 through May 16, 2020, were included. Patients without detailed information about initial symptoms were excluded. As of May 16, 2020, 795 COVID‐19 cases were confirmed in the ten prefectures. Eighty‐eight patients without detailed information about initial symptoms were excluded, and 707 patients were included in the final analysis (supplementary file: Table S2).

### 2.2 Data collection and outcome measures

Information on age, sex, symptoms, time to diagnosis from symptom onset, and the presence of pneumonia were extracted from the municipalities’ reports. The primary outcome was initial symptoms, defined as all symptoms reported on the day of symptom onset. Symptoms reported from onset until COVID‐19 diagnosis were also collected. If a patient had multiple symptoms, all the symptoms were counted.

### 2.3 Statistical analysis

The characteristics of the included patients were reported by using descriptive statistics. The proportions of patients with each individual symptom among the symptomatic patients were calculated. The proportions of patients with gastrointestinal symptoms without respiratory symptoms and those of patients with only the loss of smell or taste as the initial symptom among the symptomatic patients were also determined.

## 3 RESULTS

**Figure 1:**
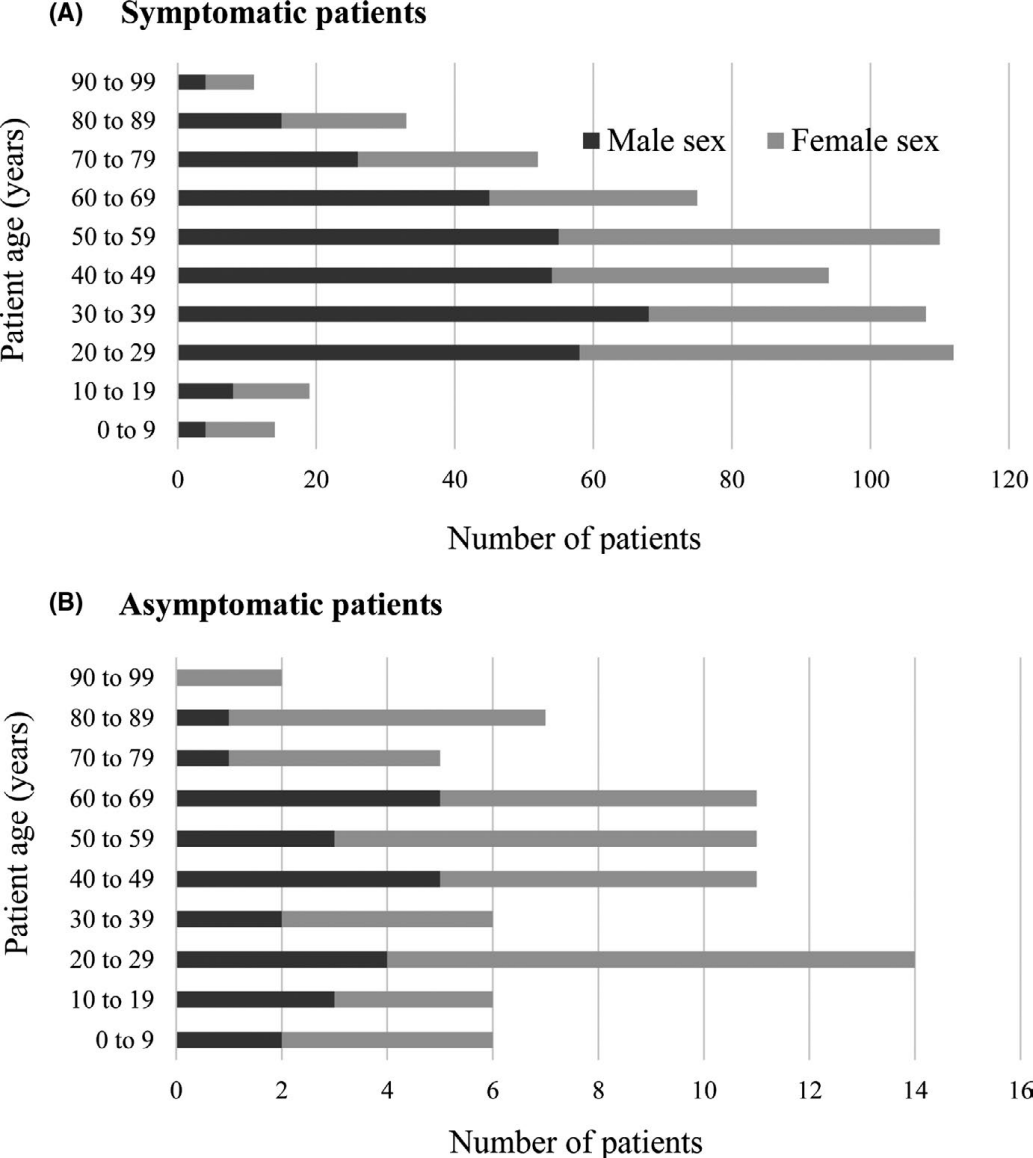
Demographic features of symptomatic (A) and asymptomatic (B) COVID‐19 patients. The total number of COVID‐19 patients stratified by age group is shown. The black bar indicates the number of men, while the gray bar indicates the number of women

Among the 707 COVID‐19 patients, 6.3% were less than 20 years old, 48.7% were women, and 11.2% were asymptomatic (Figure 1 and Table S3). Forty‐four patients (7.9%) were diagnosed with pneumonia before being diagnosed with COVID‐19. Among 627 symptomatic patients, the median days from onset to diagnosis was 6 (IQR 4 to 9). The most common initial symptom on the day of onset was fever (n = 410, 65.9%), followed by cough (n = 148, 23.5%), malaise (n = 148, 23.5%), and sore throat (n = 81, 12.9%) (Table 1). One of these four symptoms was reported in 88.2% of all symptomatic patients. Nineteen patients (3.0%) reported gastrointestinal symptoms without respiratory symptoms, while six patients (1.0%) reported only the loss of smell or taste as the initial symptom.

**Table 1:** Symptoms of the 628 symptomatic COVID‐19 patients in the initial phase

	At onset^a^	Until diagnosis^b^
Individual symptoms^c^		
Any fever	410 (65.3)	523 (83.3)
More or 38.0°C	215 (34.2)	317 (50.5)
37.0 to 38.0°C	195 (31.1)	206 (32.8)
Cough	148 (23.6)	231 (36.8)
Malaise or fatigue	148 (23.6)	215 (34.2)
Sore throat	81 (12.9)	112 (17.8)
Headache	78 (12.4)	128 (20.4)
Rhinorrhea	53 (8.4)	83 (13.2)
Arthralgia	43 (6.9)	64 (10.2)
Chills	33 (5.3)	36 (5.7)
Nasal obstruction	23 (3.7)	39 (6.2)
Throat discomfort	22 (3.5)	29 (4.6)
Loss of smell	21 (3.3)	63 (10.0)
Diarrhea	20 (3.2)	38 (6.1)
Muscle ache	20 (3.2)	29 (4.6)
Sputum	17 (2.7)	42 (6.7)
Loss of taste	14 (2.2)	57 (9.1)
Anorexia	14 (2.2)	31 (4.9)
Nausea or vomiting	14 (2.2)	28 (4.5)
Dyspnea	9 (1.4)	41 (6.5)
Abdominal pain	4 (0.6)	10 (1.6)
Chest pain	2 (0.3)	12 (1.9)
Any of the four common symptoms^d^	554 (88.2)	596 (94.9)
Any respiratory tract symptoms	264 (42.0)	368 (58.6)
Only loss of smell or taste	6 (1.0)	3 (0.5)
Gastrointestinal symptoms without respiratory symptoms^e^	19 (3.0)	26 (4.1)

Abbreviation: COVID‐19, coronavirus disease 2019.

^a^ All symptoms were reported on the day of symptom onset.

^b^ All symptoms were reported prior to the diagnosis of COVID‐19 from symptom onset.

^c^ Patients could have more than one symptom.

^d^ These include patients with fever, cough, malaise, or sore throat.

^e^ These include patients with gastrointestinal symptoms (diarrhea, abdominal pain, anorexia, or nausea) but not respiratory symptoms.

The most common symptoms reported during the median six days from onset until diagnosis were fever (n = 523, 83.3%), cough (n = 231, 36.8%), malaise (n = 215, 34.2%), and sore throat (n = 112, 17.8%). None of the four symptoms appeared in 32 patients (5.1%). Although loss of smell or taste was uncommon as the initial symptom, it developed before the diagnosis of COVID‐19 in a substantial proportion of patients.

## 4 DISCUSSION

The present study showed that fever, cough, malaise, and sore throat were the most common initial symptoms of COVID‐19 in Japan. Approximately 90% of patients reported one of these four symptoms as an initial symptom, and gastrointestinal symptoms without respiratory symptoms were uncommon. Our findings are consistent with those of previous Chinese studies ^3^, ^4^, ^5^, ^6^ showing that fever, cough, malaise, and sore throat were common but gastrointestinal symptoms were uncommon in COVID‐19 patients. However, our results are not consistent with those of a previous study^8^ reporting that approximately 40% of COVID‐19 patients present with gastrointestinal symptoms. Additional studies are needed to determine the frequency of gastrointestinal symptoms among COVID‐19 patients.

Our result is consistent with that of a previous study showing that anosmia developed after the appearance of other symptoms in some COVID‐19 patients.^9^ However, the prevalence of dysgeusia or anosmia in this study was much lower than those in previous studies^7^, ^9^ investigating hospitalized COVID‐19 patients, while the prevalence of dysgeusia or anosmia in this study was similar to that of another study^10^ investigating community COVID‐19 patients with mild symptoms. Differences in the target populations and methods of symptom assessment might have caused the discrepancies in the results between these studies.

Several limitations must be mentioned. First, a limitation of this study is that the data from municipalities’ reports were used, which is considered secondary information. Therefore, the collected data might be inaccurate. Second, we excluded 11% of all COVID‐19 cases confirmed in these prefectures due to a lack of detailed information about their initial symptoms. Therefore, selection bias might have affected our results. Third, information on symptoms that developed after diagnosis and prognosis is lacking. Fourth, the PCR tests for SARS‐CoV‐2 are not perfectly accurate for the diagnosis of COVID‐19. Moreover, patients who were clinically diagnosed with COVID‐19 but were not confirmed to have COVID‐19 by the PCR test for SARS‐CoV‐2 were not included. Finally, given that SARS‐CoV‐2 may mutate quickly, our findings are applicable only prior to the date of this study.

## 5 CONCLUSIONS

As in other countries, the most common initial symptoms of COVID‐19 patients in Japan are fever, cough, malaise, and sore throat. Gastrointestinal symptoms without respiratory symptoms are less common as initial symptoms of COVID‐19 patients in Japan.

## CONFLICT OF INTEREST

None.

## Supporting information

Supplementary Material

Supplementary Material
