# Supplementary material for: Initial symptoms of patients with coronavirus disease 2019 in Japan: A descriptive study
Source: J Gen Fam Med. 2020 Sep 26;22(1):61–4. doi: 10.1002/jgf2.378 (PMC7849337; doi:10.1002/jgf2.378)
Supplement: Supplementary file 2 — Supplementary Material [file JGF2-22-61-s002.docx]

**Figure S1.** Map of Japan. Japan consists of 47 prefectures. The prefectures in the eastern region of Japan were investigated. The ten prefectures colored in red were included in this study (Aomori, Akita, Yamagata, Miyagi, Fukushima, Niigata, Tochigi, Gunma, Ibaraki, and Yamanashi). The five prefectures colored in blue were excluded because detailed information on the initial symptoms was not publicly available for most cases. As of May 16, 2020, no COVID-19 patients were reported in Iwate prefecture (in green).

**Table S1.** Web address of the publicly available data for each prefecture. ^a^Last accessed on 31 July, 2020.

**Table S2.** The number of the COVID-19 patients included in the study from each prefecture. ^a^These patients were excluded because detailed information on the initial symptoms was not available in the reports from the prefectures.

**Table S3.** Demographic features of the symptomatic and asymptomatic COVID-19 patients. COVID-19, coronavirus disease 2019; IQR, interquartile range; NA, not applicable.
